# Kinetic analysis reveals the diversity of microscopic mechanisms through which molecular chaperones suppress amyloid formation

**DOI:** 10.1038/ncomms10948

**Published:** 2016-03-24

**Authors:** Paolo Arosio, Thomas C. T. Michaels, Sara Linse, Cecilia Månsson, Cecilia Emanuelsson, Jenny Presto, Jan Johansson, Michele Vendruscolo, Christopher M. Dobson, Tuomas P. J. Knowles

**Affiliations:** 1 Department of Chemistry, University of Cambridge, Lensfield Road, Cambridge CB2 1EW, UK; 2 Department of Biochemistry and Structural Biology, Lund University, Box 124, SE221 00 Lund, Sweden; 3 Center for Alzheimer Research, Division for Neurogeriatrics, Department of Neurobiology, Care Sciences and Society, Karolinska Institutet, S-141 57 Huddinge, Sweden

## Abstract

It is increasingly recognized that molecular chaperones play a key role in modulating the formation of amyloid fibrils, a process associated with a wide range of human disorders. Understanding the detailed mechanisms by which they perform this function, however, has been challenging because of the great complexity of the protein aggregation process itself. In this work, we build on a previous kinetic approach and develop a model that considers pairwise interactions between molecular chaperones and different protein species to identify the protein components targeted by the chaperones and the corresponding microscopic reaction steps that are inhibited. We show that these interactions conserve the topology of the unperturbed reaction network but modify the connectivity weights between the different microscopic steps. Moreover, by analysing several protein-molecular chaperone systems, we reveal the striking diversity in the microscopic mechanisms by which molecular chaperones act to suppress amyloid formation.

## Abstract (web summary)

Molecular chaperones are recognized to interfere with protein aggregation, yet the underlying mechanisms are largely unknown. Here, the authors develop a kinetic model that reveals the variety of distinct microscopic mechanisms through which molecular chaperones act to suppress amyloid formation.

## Graphical abstract

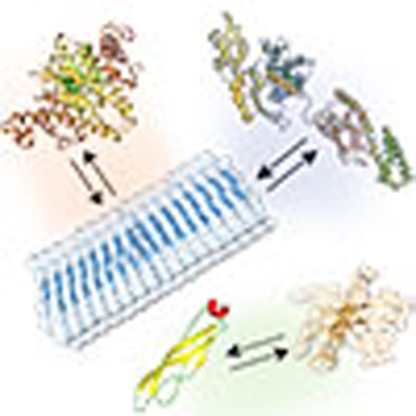

The great variety of cellular functions on which life depends reflects a multiplicity of carefully regulated interactions between macromolecules, notably involving proteins and their substrates and cofactors. Understanding the mechanisms underlying such interactions is a fundamental objective of molecular biology, not least because it opens up therapeutic opportunities of correcting dysfunctional cellular behaviours linked to disease. For example, in the context of the malignant growth of tumours, enzymes involved in cell signalling, particularly kinases, are widely investigated as targets for intervention and for the design of drug discovery programmes. Another intriguing example of dysfunctional cellular behaviour involves the pathogenic aggregation of normally soluble peptides and proteins into aberrant insoluble species known as amyloid fibrils^1^. Genetic and physiological evidence indicates that this process of self-assembly is a crucial upstream event associated with the onset and the progression of many neurodegenerative disorders, including conditions such as Alzheimer's and Parkinson's diseases^2^,^3^,^4^,^5^.

Molecular chaperones are vital components of the protein quality control system, which assist in the synthesis, folding, trafficking and degradation of proteins^6^,^7^,^8^. In addition to these roles, increasing evidence from *in vitro* and *in vivo* studies in the last decade have demonstrated that molecular chaperones play important roles in the specific suppression of amyloid formation and of the toxicity that is associated with this process^8^,^9^,^10^,^11^,^12^,^13^,^14^,^15^. Several possible mechanisms for such action have been proposed but the identification of specific molecular events associated with given protein-chaperone systems is a particularly challenging objective.

The challenge arises at a fundamental level from the large variety of potential interactions between a given molecular chaperone and the different protein species that can be present during the aggregation process (Fig. 1). Indeed, it is now clear that molecular chaperones can interact not only with monomeric misfolded or unfolded forms of proteins but also with a variety of aggregated species^16^,^17^,^18^. As a consequence, any of the microscopic steps that make up the macroscopic protein aggregation process of a given protein can be influenced by chaperone binding (Fig. 1). The identification of specific steps perturbed by an individual molecular chaperone is complicated by the heterogeneity of the various species and by the highly nonlinear nature of the protein aggregation kinetics; the latter arises from the fact that the kinetics are the result of a combination of a range of microscopic reactions involving primary and secondary nucleation, fibril growth and other secondary phenomena such as fibril fragmentation^19^,^20^,^21^,^22^,^23^,^24^,^25^.

An established route to interrogate conventional chemical systems to define reaction mechanisms consists of performing kinetic experiments and comparing the results to rate laws derived from canonical molecular mechanisms^2^,^26^. Here, we have explored the potential of this conventional workflow of chemical kinetics to the problem of understanding the mechanism of action of molecular chaperones in the context of protein misfolding and aggregation. The macroscopic kinetic profiles of fibril formation, in the case of systems containing a fixed amount of monomeric precursor proteins, follow a characteristic sigmoidal curve, where a lag-phase precedes rapid growth until a final plateau resulting from monomer depletion is observed^2^,^26^. In typical studies, the inhibitory effect of molecular chaperones can be rapidly evaluated by monitoring the overall kinetics of macroscopic aggregation, for example, by means of amyloid-specific dyes such as thioflavin T (ThT), where the fluorescence reading reports on the quantity of converted monomers. This approach has the advantage of providing rapid information on the effect of specific components on the inhibition of the overall kinetics. With this approach, however, the detailed mechanism by which fibril formation is inhibited is not immediately evident; for example, kinetic analysis shows that the lag-phase is generally strongly associated with secondary processes in addition to primary nucleation events^19^,^20^,^27^,^28^. Changes in the lag-phase cannot, therefore, be attributed simply to the influence of a given molecular chaperone on the primary nucleation process. Indeed, fibrils are observed even in the early stages of the aggregation process, and can potentially catalyse secondary reactions which generate new nuclei, thereby accelerating the reaction in an auto-catalytic way^27^. Despite these challenges, it is crucial to define strategies that allow the specific microscopic steps affected by a given chaperone to be determined in quantitative details.

In this work, we demonstrate a strategy based on chemical kinetics that is designed to go beyond the initial documentation of the inhibitory effects of particular species and their magnitudes. In this approach, the mechanism of action of a given molecular chaperone is defined in precise terms including: (1) identifying the species with which it interacts, (2) the specific microscopic processes that are perturbed, and (3) the extent to which the rate constants of the distinct steps are affected. The core of the strategy consists in building on a previous kinetic approach to develop a new model that describes pairwise interactions between molecular chaperones and the different protein species. We show that these interactions conserve the topology of the unperturbed aggregation network and modify the connectivity weights between the different microscopic reaction steps (Fig. 1). We apply this framework to analyse the mechanisms of inhibition by a set of molecular chaperones of the processes of the formation of amyloid fibrils by different proteins, thereby providing a comprehensive mechanistic picture of the modes of action by which molecular chaperones act to suppress amyloid fibril formation.

## Results

### Microscopic mechanisms from macroscopic kinetic profiles

The formation of protein aggregates, such as amyloid fibrils, is the consequence of a series of microscopic events, including primary nucleation, fibril elongation, fibril fragmentation and secondary nucleation processes, which can be associated with reaction rate constants *k*_*n*_, *k*_+_, *k*_−_ and *k*_2_, respectively^2^,^26^. The time evolution of fibril formation can be described by a master equation that includes the contributions of the various individual microscopic processes (Supplementary Equations 1–3)^21^,^29^,^30^ Self-consistent solutions of the master equation provide compact expressions for the time evolution of the total fibril mass concentration (*M*) of the type^21^,^29^,^30^:


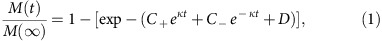


where the kinetic parameters *C*_±_, *D* and *κ* are functions of a limited number of combinations of the microscopic rate constants: *k*_*n*_*k*_+_, *k*_*n*_/*k*_−_ and *k*_2_*k*_+_ (see Supplementary Equation 4 for details). These expressions have been shown to describe succesfully the kinetic profiles observed for protein aggregation reactions under a wide range of conditions, and to identify the relative contributions of the single microscopic events to the global aggregation rate^26^.

In the presence of a molecular chaperone, the kinetics of aggregation may be altered as a result of the interactions between the molecular chaperone and one or more of the protein species present in the system, which can lead to fundamental changes in the microscopic events involved in the aggregation process. In this work we build on the insight that Equation 1 can be used to interpret the effects of inhibitors on the reaction kinetics and thus to obtain information on such microscopic events from the global aggregation profiles. The key to applying this approach is to note that changes in different microscopic events result in different characteristic changes in the macroscopic aggregation profiles, as shown in the model simulations in Fig. 2a–c. For example, a decrease in the primary nucleation rate will simply increase the lag-time preceding the growth phase, but decreases in the elongation rate or in the number of secondary nucleation events will affect both the lag-phase and the growth phase, but in a different and characteristic manner. We can quantify in a semi-empirical way the effect of a molecular chaperone on the different individual steps of the reaction by describing the experimentally measured aggregation kinetic curves using Equation 1, and perturbing in a systematic manner the sets of microscopic kinetic rate constants, *k*_*n*_*k*_+_, *k*_*n*_/*k*_−_ and *k*_2_*k*_+_, evaluated in the absence of the molecular chaperone. In this way, the modification of the reaction rates induced by the presence of a molecular chaperone can be described by introducing suitable apparent reaction rate constants. The comparison between the sets of kinetic rate constants defined in the absence and presence of different concentrations of each molecular chaperone provides quantitative information on the microscopic processes that are affected by its presence.

We illustrate the potential of this analysis by considering examples of the inhibition of specific molecular chaperones on the aggregation kinetics of two different amyloidogenic systems (Fig. 2). The first example refers to A*β*42, a peptide whose aggregation is linked to Alzheimer's disease, in the presence of a molecular chaperone belonging to the DNAJ family (DNAJB6)^31^. As shown in Fig. 2d, in this system the analysis of the reaction profiles at different chaperone concentrations reveals that under these conditions the presence of the chaperone results mainly in the inhibition of the primary nucleation process.

The second example (Fig. 2e) involves the aggregation of the yeast prion protein Ure2p in the presence of Ssa1, a molecular chaperone belonging to the Hsp70 family^32^. We find that the aggregation kinetics at different chaperone concentrations can be well described by Equation 1 by modifying only the elongation rate constant (*k*_+_), keeping constant both the fragmentation (*k*_−_) and the nucleation rate constant (*k*_*n*_). This result indicates clearly that in this case the molecular chaperone inhibits specifically the elongation process.

In the last example, shown in Fig. 2f, we illustrate the inhibition by proSP-C Brichos, a molecular chaperone belonging to the Brichos family, of the aggregation of A*β*42 (ref. 33). The kinetic profiles at different chaperone concentrations indicate that the presence of proSP-C Brichos results in a specific reduction in the secondary nucleation rate, indicating that the chaperone inhibits the catalytic activity of the fibril surface. The decrease of the different microscopic rate constants as a function of the chaperone concentration is shown in the inserts of Fig. 2d–f.

### Development of kinetic model describing binary interactions

**Figure 1:**
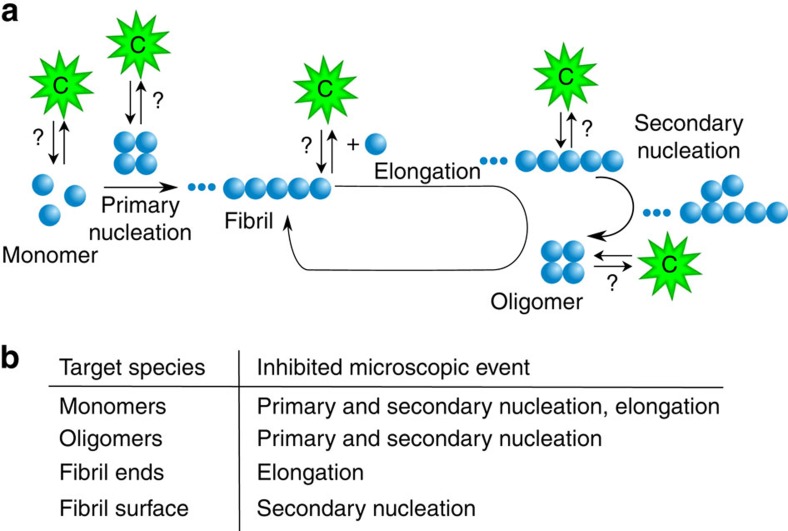
Schematic illustration of an aggregation reaction network. (**a**) This network is made up by the microscopic steps in the aggregation mechanism of an amyloidogenic protein (blue), and the several possible interactions between a molecular chaperone (green) and the different protein species present in the system; (**b**) Depending on the target species, different microscopic steps can be affected.

Systems of aggregating proteins commonly contain a heterogeneous population of protein species ranging from monomers to soluble oligomers and mature fibrils^2^,^26^. In the following sections, we define a monomer as the aggregation-competent unit, and an aggregate as any species composed of several aggregating units. We note that this definition includes oligomers as aggregates. However, considering the current challenges in the experimental characterization of the structure and function of the oligomers, these species will not be considered in the mathematical treatment described here. In principle, a molecular chaperone can suppress one or more different microscopic reactions by interacting with one or more of the species present in the system (Fig. 1a). The identification of the microscopic step perturbed by the molecular chaperone already provides some insights into the likely target species, as illustrated in Fig. 1b. Indeed, interactions with monomers cause a decrease in the rates of several microscopic steps, since these monomeric species participate in many of the key molecular level events, in particular primary and secondary nucleation in addition to elongation. Interactions with oligomers, defined in this specific context as intermediates in the aggregation process leading to fibril formation independently of their morphology, can reduce primary and secondary nucleation rates, while binding to the fibril ends will suppress only the elongation process, and binding along the fibril surfaces will decrease the surface catalysed secondary nucleation rate^2^,^26^,^34^. Thus, for example, in the system involving A*β*42 and the molecular chaperone DNAJB6, the identification of the selective inhibition of primary nucleation together with stoichiometric considerations indicate that the mode of action of this molecular chaperone in the system involves interactions with the growing aggregates rather than with the monomers^31^.

Molecular chaperones can sequester misfolded monomers or oligomers in solution, or bind to fibril surfaces or fibril ends, as illustrated in Fig. 1. Each of those events changes the concentration of one or more of the reactive species in the system. We can build further on the kinetic platform to identify the target species interacting with the molecular chaperones by introducing in the master equation, the contributions from the association and dissociation reactions of the molecular chaperones and the different target species. For instance, in the case of binding between the molecular chaperones and monomeric species (Fig. 1a), the mass balance equation governing the time evolution of the soluble monomers (*m*(*t*)) is:


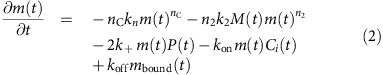


where *C*_*i*_ the concentration of the free binding sites of the molecular chaperones, and *m*_*bound*_ is the concentration of monomers bound to the molecular chaperones.

Binding to soluble monomers reduces their free concentration, thereby decreasing the rates of all nucleation steps and the elongation processes. In analogy with the situation in the absence of inhibitors^21^,^29^,^30^, an analytical treatment of the mass balance equation based on the perturbative renormalization group^35^ leads to the the derivation of closed-form expressions for the time evolution of the fibril mass. For instance, the application of this analytical treatment to Equation 2 (Supplementary Methods) provides the following closed form:


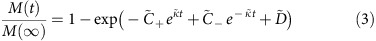


where the constants 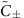, 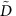 and 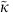 depend on specific combinations of the microscopic rate constants of filamentous growth and on the mechanistic details of the reversible association reaction, including the equilibrium binding constant *K*_*eq*_ and the equilibrium concentration of free chaperone (see Supplementary Equations 37–51 for details).

Analogous equations for the mass balances and the closed analytical forms of the total mass and number concentration of fibrils can be derived in the case of binding to fibril ends or fibril surfaces using the same techniques, as discussed in the Supplementary Methods.

With respect to the situation in the absence of molecular chaperones, in addition to the set of rate constants described in the aggregation process, the only additional parameters required to describe the kinetic data are the association and dissociation rate constants. Fitting to experimental data, therefore, provides estimates of the association and dissociation rate constants in solution, which can be compared with the values measured independently by other experimental techniques, such as biosensors.

### Examples of specific inhibition

**Figure 2:**
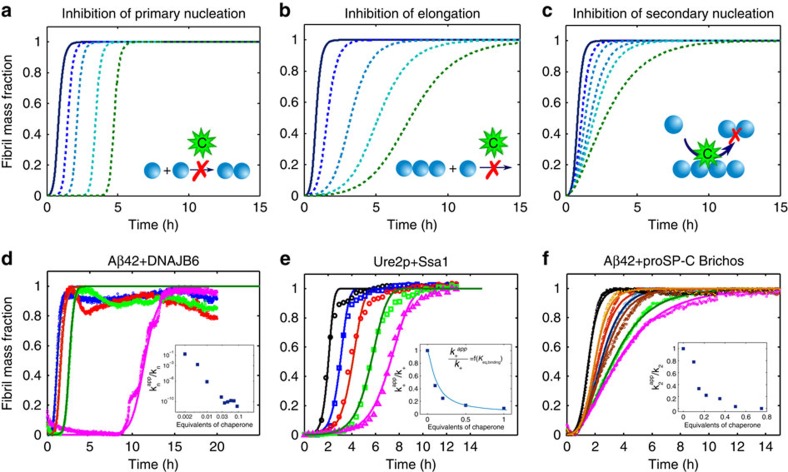
Molecular chaperones can affect individual microscopic steps in the aggregation process. (**a**–**c**) Numerical integration of the master equations illustrates how perturbations of specific microscopic aggregation events modify in characteristic ways the global kinetic profiles. The analysis of the changes in the macroscopic profiles provides quantitative information on the microscopic events altered by the molecular chaperones. (**d**–**f**) To demonstrate the experimental consequences of the different mechanisms described in (**a**–**c**) we consider the aggregation kinetics of the A*β*42 peptide and the prion protein Ure2p of in the absence and presence of different molecular chaperones at increasing concentrations. (**d**) DNAJB6 inhibits strongly the primary nucleation rate of unseeded 3 μM A*β*42 aggregation reactions in phosphate buffer pH 8.0 at 37 °C (ref. 31); (**e**) a member of the Hsp70 family (Ssa1) suppresses specifically the fibril elongation rate in the aggregation process of 30 μM Ure2p in Tris buffer pH 7.5 at 30 °C (ref. 32); (**f**) a molecular chaperone belonging to the Brichos family (proSP-C Brichos) inhibits specifically the secondary nucleation rate in the aggregation of 3 μM A*β*42 in phosphate buffer pH 8.0 at 37 °C (ref. 33). The molecular equivalents of chaperones as well as the microscopic rate constants as a function of molecular chaperone concentration, evaluated by fitting the experimental data with Equation 1, are shown in the inserts. The continuous line in the inset in (**e**) corresponds to the evaluation of the binding equilibrium constant according to Equation 4.

**Figure 3:**
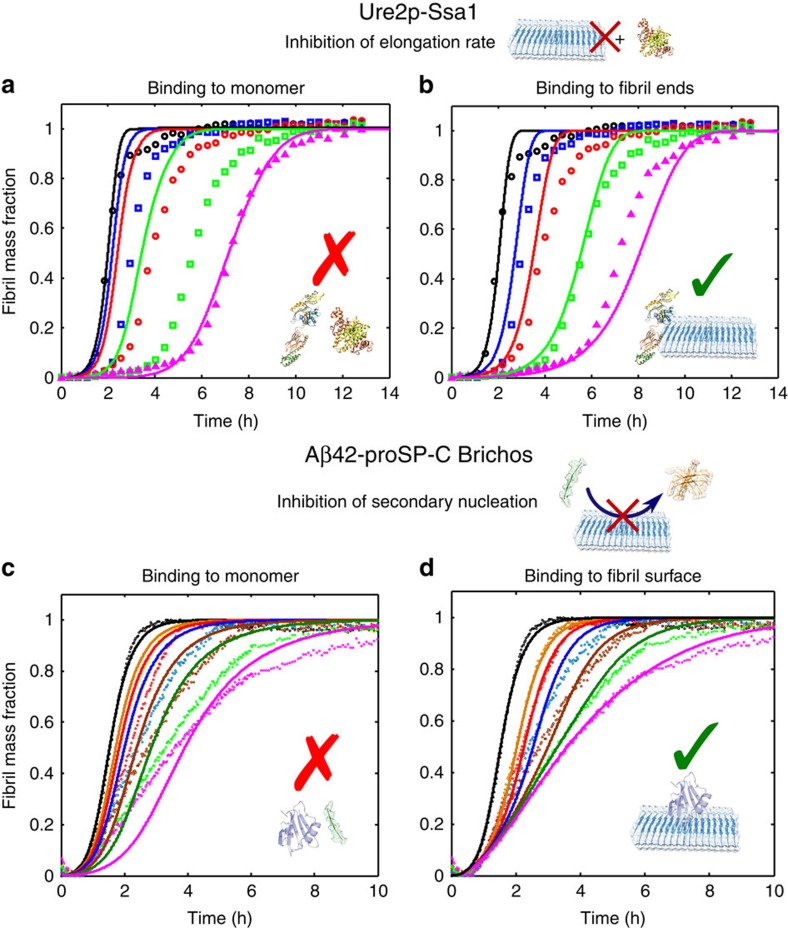
Identification of the target species interacting with different molecular chaperones. Two specific sets of data taken from the literature^32^,^33^ are re-analysed in the frame of the novel kinetic model developed here. The reaction profiles at different molecular chaperone concentrations are compatible with binding to the fibril ends in the case of the Ure2p-Saa1 system (**a**,**b**) and to the fibril surface in the case of the A*β*42-Brichos system (**c**,**d**). The global fitting was performed by applying the set of equations reported in the Supplementary Equations 5–24.

We demonstrate the potential of the kinetic model by analysing two particular examples reported in the literature in the frame of the novel approach developed in this work. First, we consider the system discussed above involving the prion Ure2p (ref. 32). The semi-empirical analysis based on Equation 1 indicates that the molecular chaperone Ssa1 inhibits specifically the elongation rate (Fig. 2e). Considering the qualitative correlation between the single inhibited processes and the target species shown in the schematic mechanism in Fig. 1, we might expect that this inhibition originates mainly from interactions between the molecular chaperone and the fibril ends, since significant interactions between the molecular chaperone and the monomer would suppress all the microscopic steps of the process, in contrast with the observation that the elongation rate is the major microscopic step that is specifically inhibited. We can therefore examine this prediction in a quantitative manner by attempting a description of the kinetic data in cases where the binding of the molecular chaperone is to fibril ends or to monomers. In Fig. 3a,b we show the best fits to the experimental data in these two cases: it can be seen that the calculations assuming binding to fibril ends describe the dependence of the global kinetic profiles on the molecular chaperone concentration much better than those assuming binding to monomer (the least-squared error function, defined in the online methods, is 0.9 for binding to fibril ends and 3.8 for binding to monomers). We can therefore conclude that the molecular chaperone does indeed interact mainly with fibril ends. From the fitting, a binding constant, *K*_*eq*_=3 × 10^5^ M^−1^ can be estimated, corresponding to a free energy of binding Δ*G*^0^=−31 kJ mol^−1^.

We next focus on the molecular chaperone proSP-C Brichos discussed above that was shown in the semi-empirical approach to inhibit A*β*42 aggregation by suppressing specifically the dominant secondary nucleation process in the system, namely the surface-induced secondary nucleation reaction (Fig. 2f) (ref. 33). As in the case of Ssa1 and Ure2p, the qualitative analysis schematized in Fig. 1 would exclude significant interactions between the molecular chaperone and the A*β*42 monomers, since the binding to these species would perturb all the microscopic steps of the process. To examine this hypothesis in more detail, we described the kinetic profiles at different molecular chaperone concentrations by considering binding of the molecular chaperone either to monomer A*β*42 or to the surfaces of fibrils (Fig. 3c,d). The best fits to the experimental data shown in Fig. 3d are calculated assuming that the molecular chaperones bind mainly to the fibril surface; binding to monomers is not compatible with dependence of the inhibiton rate on the molecular chaperone concentration (the least-squared error function, defined in the online methods, is 1.5 for binding to the fibril surface and 8.1 for binding to monomers). A very strong contribution of the kinetic model developed here comes from the finding that the values of association and dissociation rate constants that are estimated in the case of binding to the fibril surface (*k*_*on*_=2 × 10^4^ M^−1^ s^−1^ and *k*_*off*_=1 × 10^−4^ s^−1^) are similar to those measured independently by surface plasmon resonance^33^ (*k*_*on*_=5 × 10^3^ M^−1^ s^−1^ and *k*_*off*_ =2 × 10^−4^ s^−1^), and only an additional minor contribution coming from the binding to fibril ends was required to describe the kinetic profiles at different chaperone concentrations. The binding constant estimated by model simulations, *K*_*eq*_=2 × 10^8^ M^−1^, corresponds to a binding free energy of Δ*G*^0^=−47 kJ mol^−1^, similar but slightly larger than the free energy evaluated in the previous system for binding to fibril ends.

### Analysis of more complex scenarios

**Figure 4:**
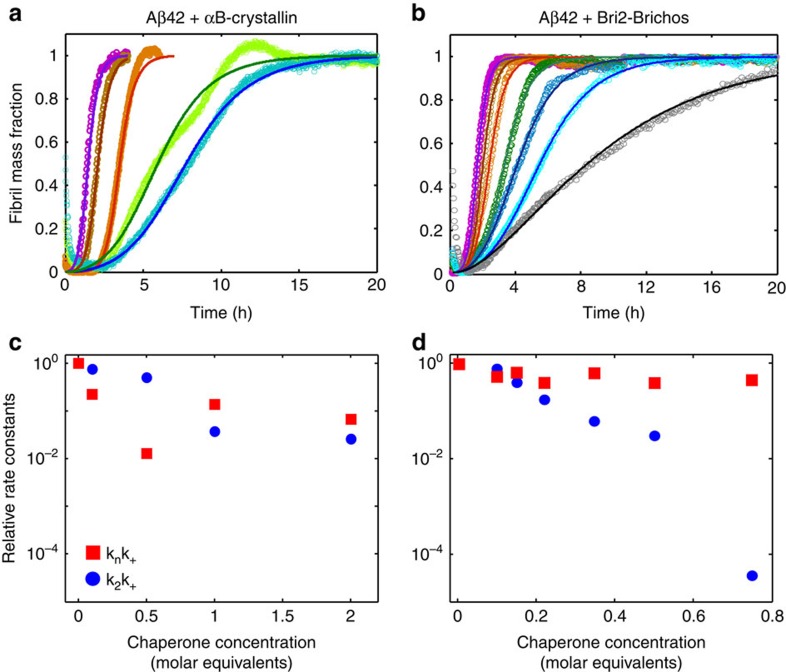
Molecular chaperones can affect simultaneously several microscopic steps in the aggregation process. Reaction profiles of the aggregation of 3 μM A*β*42 in the absence and presence of (**a**) *α*B-crystallin and (**b**) a member of the Brichos family (Bri2-Brichos) in phosphate buffer pH 8.0 at 37 °C. The continuous lines represent the integrated rate laws. The molecular equivalents of chaperones as well as the microscopic rate constants as a function of the molecular chaperone concentration relative to A*β*42 are reported in (**c**) and in (**d**) relative to the values in the absence of the molecular chaperone.

The examples described above represent cases where a molecular chaperone modulates mainly a single microscopic event. In other more complex situations, a molecular chaperone can influence several microscopic processes simultaneously. Two examples of such a situation are reported in Fig. 4, where we show the aggregation profiles of A*β*42 in the absence and presence of the molecular chaperone *α*B-crystallin as well as another member of the Brichos family (Bri2-Brichos). The kinetic analysis shows that these two molecular chaperones affect different microscopic processes involved with the aggregation of the A*β*42 peptide. In particular, this analysis, together with experimental evidence of the binding of the molecular chaperones to the aggregates^16^, indicates that the molecular chaperones are able to modulate both elongation and secondary nucleation processes.

Another remarkable implication of the kinetic analysis is concerned with the topology of the reaction network in the presence of biological perturbations. The reaction network can be defined as the series of microscopic events which contribute to the overall aggregation mechanism, and the topology of the network describes the way through which these different microscopic processes are connected (Fig. 1a). The presence of molecular chaperones could potentially interfere with the formation of amyloid fibrils by a large variety of mechanisms, including not only the change in the relative contribution of the individual microscopic steps to the global aggregation mechanism, but also the fundamental modification of the nucleation-polymerization pathway. For instance, interactions with molecular chaperones could induce structural rearrangements of the aggregating species or additional aggregation reactions leading to amorphous species. The strong agreement between model simulations and experimental data acquired at different chaperone concentrations (Fig. 3b,d) shows that in several systems the aggregation network observed in the absence of biological perturbations is conserved in the presence of molecular chaperones, and the interactions between the molecular chaperones and the different protein species modify the connectivity weights between the different microscopic steps (that is, the relative contribution of the individual reactions to the overall aggregation mechanism) without affecting the topology of the network. This observation explains why the semi-empirical approach based on Equation 1 is capable of describing the modifications of the kinetic profiles observed in the presence of molecular chaperones by suitable perturbations of the rate constants of specific microscopic reactions of the network. Moreover, for the systems investigated in this work, imaging techniques confirm that aggregates of fibrillar morphology are formed both in the absence and in the presence of molecular chaperones^31^,^32^,^33^. Our model could be further improved by taking into consideration additional microscopic events, such as lateral aggregation between fibrils, which could be responsible for the changes in the plateau values of the raw ThT profiles which are observed in certain systems in the presence and in the absence of molecular chaperones. These additional microscopic events, however, appear to contribute only marginally to the inhibition mechanism with respect to the processes considered in this work.

In addition, we note that the potential alternative reaction mechanisms with respect to nucleation-polymerization models could be captured by the kinetic approach based on population balance equations discussed here, provided that sufficient experimental information is available to enable fitting of the data to a given model.

### Quantification of interaction energies from reaction rates

In situations where the association and dissociation rates of binding of the molecular chaperone are much faster than the microscopic aggregation reactions of the peptide or protein, the system can be considered to be at equilibrium, and we can derive here simple expressions for the effective microscopic reaction rates as a function of molecular chaperone concentration by analogy with the closed expressions obtained in the context of the inhibition of enzymatic catalysis in the presence of ligands^36^.

For example, in the case of binding to fibril ends, the effective elongation rates can be readily derived as (Supplementary Equations 30–34):


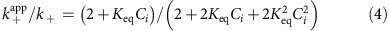


where *k*_+_ is the elongation rate constant in the absence of inhibitor, *K*_*eq*_ the equilibrium binding constant and *C*_*i*_ the free chaperone concentration, which can be approximated as the total concentration of molecular chaperone in the case of a large excess of the latter relative to the number of fibril ends. For the system involving the prion Ure2p and the molecular chaperone Ssa1 (inset of Fig. 2e), we compare the apparent values of the elongation rate constants estimated by the fits shown in Fig. 2 with those predicted by Equation 4. The two sets of values are in exact agreement and, as expected, the value of *K*_*eq*_ required to describe the apparent elongation rate constants with Equation 4 is equal to the equilibrium constant evaluated by the integrated rate laws shown in Fig. 2. This observation emphasizes the fact that, in systems where the binding process can be considered to be under equilibrium conditions, the apparent kinetic constants evaluated by the semi-empirical approach based on Equation 1 can provide direct measures of the equilibrium binding constant (*K*_*eq*_) and of the free energy of binding (Δ*G*^0^).

## Discussion

Increasing evidence from *in vitro* and *in vivo* studies indicates that, in addition to their role in assisting protein folding^6^,^7^,^8^, molecular chaperones are able to influence very significantly the specific formation and the kinetic stability of amyloid fibrils^8^,^9^,^10^,^11^,^12^,^13^,^15^. *In vitro* studies suggest that these effects could be the consequence of several possible interactions not only with the monomeric species but also with the aggregated forms of the misfolded proteins^16^,^17^,^18^. The identification of the specific interactions occurring in the heterogeneous population of protein species, however, has been proved to be challenging to achieve using biochemical techniques. To address this problem, in in this work we describe a framework based on chemical kinetic analysis to elucidate the microscopic mechanisms underlying the modulation of amyloid formation by molecular chaperones. The key of this strategy is represented by the development of a novel kinetic model that describes pairwise interactions between molecular chaperones and the different protein species. The comparison between model simulations and experimental data acquired at different chaperone concentrations provides a series of remarkable findings. First of all, the agreement between simulated and experimental kinetic profiles indicates that the interactions between molecular chaperones and the different protein species modify the connectivity weights between the different microscopic steps without affecting the topology of the aggregation network observed under unperturbed conditions. Moreover, the strategy allows the identification of both the specific protein species that interact with a given molecular chaperone and the microscopic aggregation steps that are affected by such an interaction. In addition, we have identified limiting situations where the evaluation of the apparent rate constants can lead to the estimation of the equilibrium constant of binding, in ways that are analogous to the well-established analysis of kinetics of enzyme inhibition^36^.

**Figure 5:**
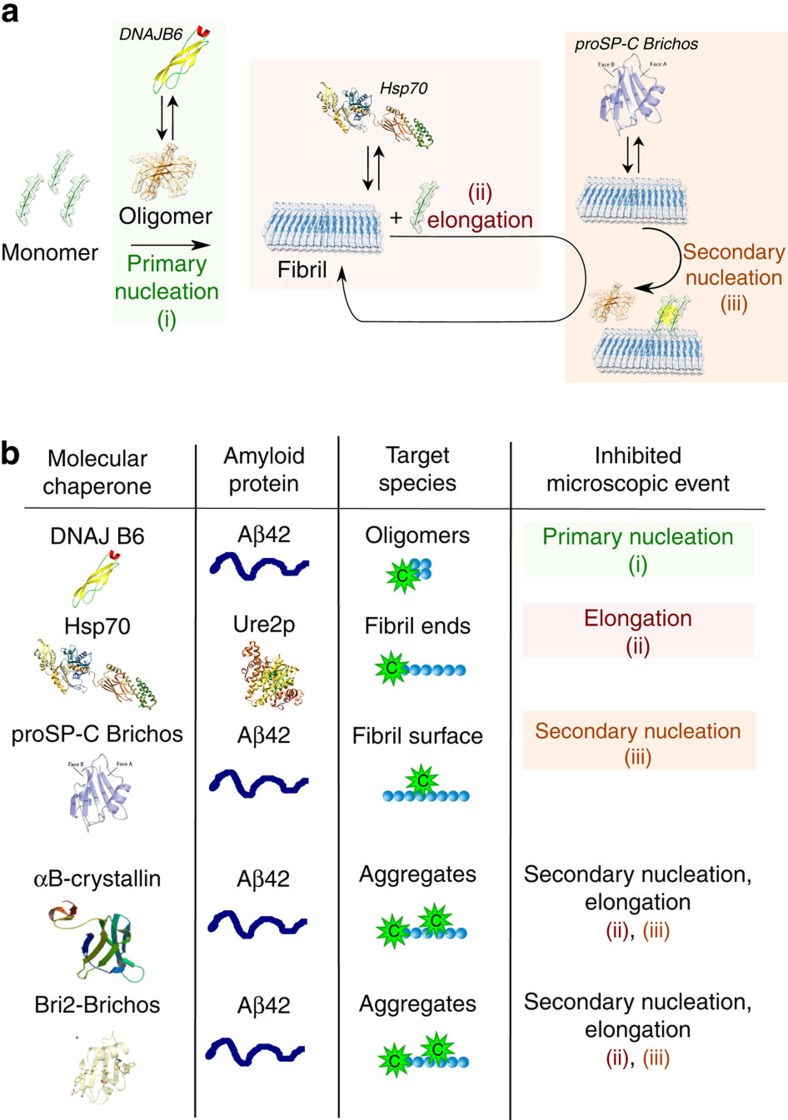
Summary of the diverse modes of action. Overview of the variety of diverse microscopic mechanisms through which molecular chaperones can suppress amyloid formation, as revealed by the kinetic analysis presented in this work. As we demonstrate, molecular chaperones have evolved to exploit in a variety of ways the different opportunities to modulate protein aggregation offered by the binding to different protein species (Fig. 1).

By applying this analysis to a set of systems involving different amyloidogenic proteins and molecular chaperones, we have provided a detailed mechanistic description of a variety of the modes of action through which molecular chaperones can suppress amyloid fibril formation. The analysis has shown that these naturally occurring inhibitors of protein aggregation are able to exert a protective function through a variety of diverse microscopic mechanisms, as summarized in Fig. 5. This analysis has also revealed that some molecular chaperones interact rather selectively with specific forms of the proteins and suppress specific aggregation steps, while others affect simultaneously different microscopic events by interacting with several species. In some cases, as in the inhibition of A*β*42 aggregation by DNAJB6, large effects are evident even at highly low sub-stoichiometric ratios of DNAJB6 to A*β*42, because the molecular chaperone interacts with small oligomeric species which are intermediates to the fibril formation. This behaviour could reflect the ability that molecular chaperones have developed to recognize specific hydrophobic patches of particular misfolded species to allow high efficiency at low concentrations.

The application of the approach described in this paper to other systems will increasingly provide more insights into the modes of action of molecular chaperones in suppressing the formation of amyloid forms of proteins and its consequences for the biological systems. These primary results, however, already indicate that molecular chaperones have evolved to prevent the formation of amyloid fibrils by developing the capacity to interact with different misfolded protein species and hence to be able to perturb all the microscopic steps of the aggregation process. The identification of the microscopic mechanisms of the action of molecular chaperones against amyloid formation is not only relevant in the context of understanding how living systems are able to suppress under normal circumstances the effects of protein misfolding and aggregation, but also in the context of the rational design of potential therapeutic strategies targeted against clinical disorders resulting from protein aggregation^34^. In addition, the kinetic framework described in this work opens up the possibility of probing at a molecular level the mechanism of action of other classes of compounds which have been shown to be able to delay the formation of amyloid fibrils^37^,^38^, including small molecules^39^,^40^,^41^, peptides^42^ and proteins^13^,^43^ as well as nanoparticles^44^,^45^,^46^.

## Methods

### Materials

Human A*β* peptide, A*β*(M1-42), UniProtKB ID P05067 and aa 672–713, with an N-terminal methionine corresponding to residue 671, (MDAEFRHDSGYEVHHQKLVFFAEDVGSNKGAIIGLMVGGVVIA) was recombinantly expressed in E. coli BL21 DE3 star PLysS and purified essentially as described previously^47^. Human *α*B-crystallin (UniProtKB ID P02511) was recombinantly expressed and purified as described previously^15^,^48^. The Bri2-Brichos chaperone domain, representing Bri2 residues 90–236 (UniProtKB ID Q9Y287), was expressed and purified as a fusion protein with an S-tag, from E. coli as described previously^49^.

### Experimental kinetic assay

Aggregation kinetics were monitored using a ThT fluorescence assay based on the enhanced quantum yield for ThT fluorescence as the dye binds to amyloid fibrils. All experiments were performed in an assay buffer (20 mM sodium phosphate buffer, pH 8.0, 0.2 mM EDTA and 0.02 % sodium azide) with 10 μM ThT in microplate wells (Microplate Corning 3881, 96 well, low binding, half area, Corning Incorporated Life Sciences, Acton, MA). Samples were prepared on ice in low binding tubes using freshly prepared A*β*42 monomer. ThT fluorescence was recorded under quiescent conditions at 37 °C, using a Fluostar Omega or Optima plate reader (BMG Labtech, Offenburg, Germany) with a 440-nm excitation filter and a 480-nm emission filter^50^. The data for the yeast Ure2p prion and the chaperone Ssa1, for A*β*42 and the DNAJB6 molecular chaperone, and for A*β*42 and the proSP-C Brichos are taken from refs 31, 32, 33, respectively.

### Kinetic model analysis

For a given chaperone-protein system, the aggregation profiles in the absence and presence of different molecular chaperone concentrations were simulated according to the procedure described in the Supplementary Methods. Briefly, for a given chaperone-protein system, the aggregation profiles in the absence and presence of *n* different chaperone concentrations *C*_*i*_ were simulated individually according to Equation 1. The microscopic rate constants were determined by fitting the experimenal data by minimizing a least-squared error function defined as 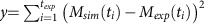, where *M*_*sim*_(*t*_*i*_) and *M*_*exp*_(*t*_*i*_) are the simulated and the experimental total fibril mass fraction at time *t*_*i*_, respectively. A similar procedure was followed to estimate the association and dissociation rate constants according to Equation 2 and the other related equations described in the Supplementary Methods. In this case, the reaction profiles at *n* different concentrations of chaperone were globally fitted by defining a least-squared error function: 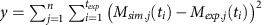, where *M*_*sim*,*j*_(*t*_*i*_) and *M*_*exp*,*j*_(*t*_*i*_) are, respectively, the simulated and the experimental total fibril mass fraction at time *t*_*i*_ at a given concentration *j* of molecular chaperone.

## Additional information

**How to cite this article:** Arosio, P. *et al*. Kinetic analysis reveals the diversity of microscopic mechanisms through which molecular chaperones suppress amyloid formation. *Nat. Commun.* 7:10948 doi: 10.1038/ncomms10948 (2016).

## Supplementary Material

Supplementary Information Supplementary Figures 1-3, Supplementary Methods and Supplementary References.
