# Supplementary material for: Kinetic analysis reveals the diversity of microscopic mechanisms through which molecular chaperones suppress amyloid formation
Source: Nat Commun. 2016 Mar 24;7:10948. doi: 10.1038/ncomms10948 (PMC4820785; doi:10.1038/ncomms10948)
Supplement: Supplementary Information — Supplementary Figures 1-3, Supplementary Methods and Supplementary References. [file ncomms10948-s1.pdf]

Supplementary Figures

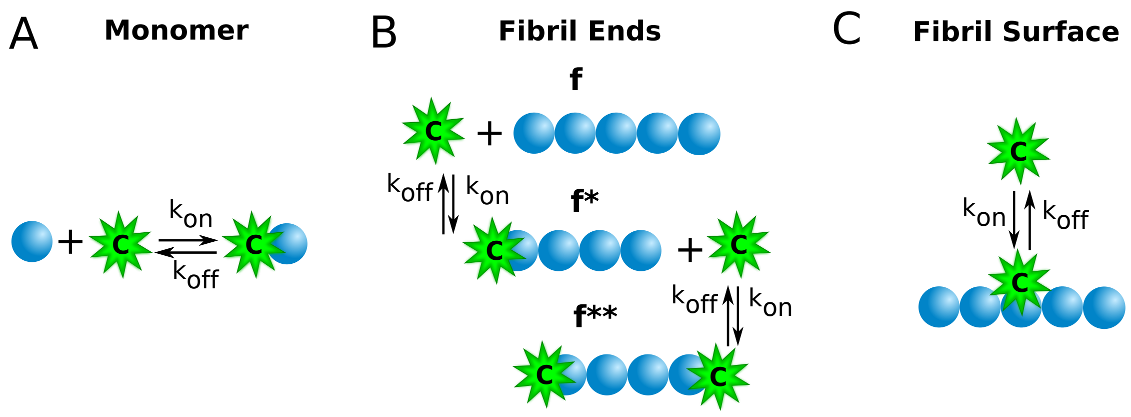

Supplementary Figure 1 – Schematization of the binding events between the molecular chaperone and the different protein species present in the aggregating system.

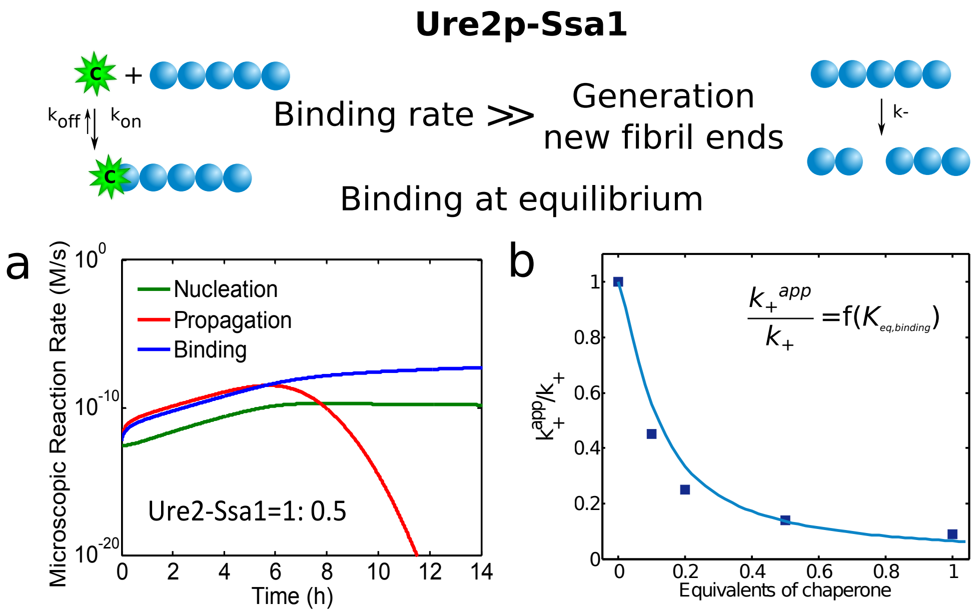

Supplementary Figure 2 – Connection between the microscopic events identified by the semi-empirical approach based on Eq.1 in the main text and the corresponding molecular targets elucidated by the more rigorous quantitative analysis which considers the binding events in the kinetic scheme. (a) In the case of the Ure2p-Hsp70 system, the binding rates are much faster with respect to the generation of fibril ends and the binding can be considered to be under equilibrium conditions. (b) A simple expression of the reduced elongation rate constant as a function of the binding constant can be derived. This reduced elongation rate constant does not change during the reaction process, and is equivalent to the value evaluated by the semi-empirical approach based on Eq. 1 in the main text.

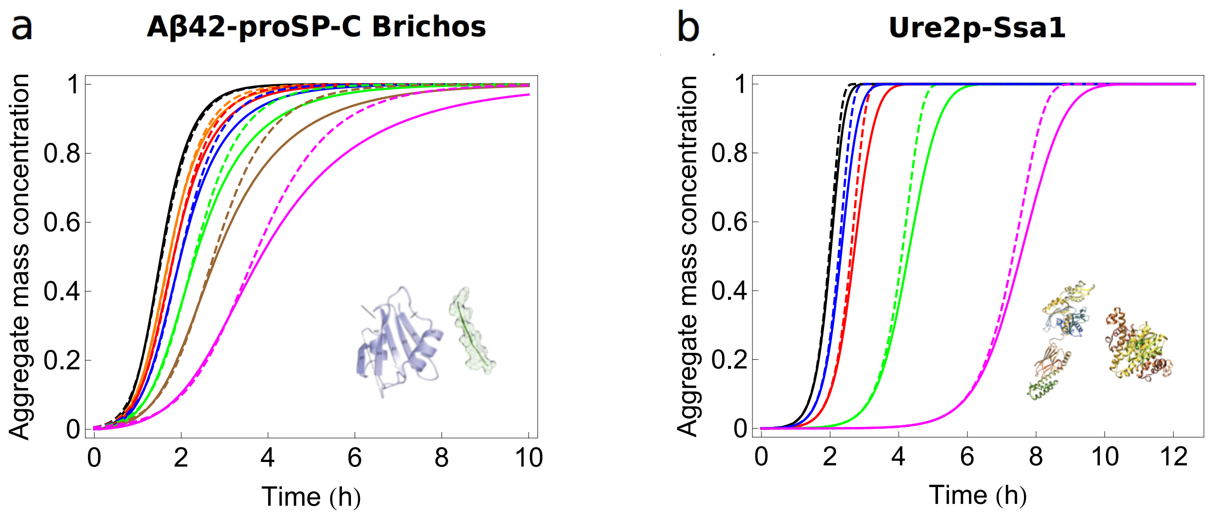

Supplementary Figure 3 – Comparison between analytical close-form solutions and numerical calculations: simulation of the time-evolution of the fibril mass fraction when the inhibition mechanism is described by binding of the molecular chaperone to the monomeric form of peptides and proteins. The examples shown are the two systems discussed in the main text: (a) the Aβ42 peptide and the Brichos chaperone, and (b) the Ure2p and the Ssa1 chaperone. For different peptide-molecular chaperone ratios, the dot lines represent the analytical close-form solutions according to Supplementary Eq. (50), while the continuous lines correspond to the numerical integration of Eq. (2) in the main text. The concentrations of amyloidogenic proteins and molecular chaperones are indicated in Fig. 2 in the main text. Kinetic parameters are: a) $k_+ = 3 \cdot 10^6$ 1/M/s, $k_n = 3 \cdot 10^{-4}$ 1/M/s, $k_2 = 1 \cdot 10^4$ 1/M^2/s^2, $n_C = 2$, $n_2 = 2$, $k_{on} = 10^3$ 1/M/s, $k_{off} = 10^{-3}$ 1/s; b) $k_+ = 8.8 \cdot 10^6$ 1/M/s, $k_n = 3 \cdot 10^{-4}$ 1/M/s, $k_- = 3.7 \cdot 10^{-6}$ 1/s, $n_C = 2$, $k_{on} = 3.7 \cdot 10^2$ 1/M/s, $k_{off} = 1.2 \cdot 10^{-3}$ 1/s.

Supplementary Methods

Kinetic model in the absence of molecular chaperone

The nucleation-elongation-fragmentation kinetics of the formation of amyloid fibrils is governed by the following master equation for the time evolution of the concentrations $f_j(t)$ of chains of length j:[1-5]

$$\begin{aligned}\frac{\partial f_j(t)}{\partial t} =& 2k_+m(t)(f_{j-1}(t) - f_j(t)) - k_-(j-1)f_j(t) + 2k_- \sum_{i=j+1}^{\infty} f_i(t) \\ &+ \delta_{j,n_C}k_n m(t)^{n_C} + \delta_{j,n_2}k_2 m(t)^{n_2} \sum_{i=n_C}^{\infty} i f_i(t)\end{aligned} \tag{1}$$

where n_C and n_2 are the reaction orders of primary and secondary nucleation, respectively, often equal to the number of monomers in the respective nuclei, δ_{j,n_i} is the Kronacker function and m is the concentration of the soluble monomer in the system. The first two terms on the right side of the equation describe the formation and the disappearance of fibrils of a given length by elongation. The following two terms describe the fragmentation events, which create smaller fragments from a longer filament, and the last two terms refer to the generation of new fibrils by primary and secondary nucleation events, respectively. By solving the master equation with the method of the moments it is possible to derive the time evolution of the total fibril number (P) and fibril mass (M) concentration:[1-4]

$$\frac{dP(t)}{dt} = k_-[M(t) - (2n_C - 1)P(t)] + k_n m(t)^{n_C} + k_2 M(t) m(t)^{n_2} \tag{2}$$

$$\frac{dM(t)}{dt} = 2[k_+m(t) - n_C(n_C-1)k_-/2]P(t) + n_C k_n m(t)^{n_C} + n_2 k_2 M(t) m(t)^{n_2} \tag{3}$$

Approximate analytical solutions of Supplementary Eq. (2) - (3) provide compact expressions for the time evolution of the total fibril mass concentration of the type:

$$\frac{M(t)}{M(\infty)} = 1 - [\exp(-C_+e^{\kappa t} + C_-e^{-\kappa t} + D)], \tag{4}$$

where the kinetic parameters $C_\pm$, D, and κ are functions of a limited number of combinations of the microscopic rate constants: k_nk_+, k_n/k_- and k_2k_+. In particular, $\kappa = \sqrt{2k_-k_+m(0)}$ or

$\kappa = \sqrt{2m(0)k_2k_+m(0)^{n_2}}$ when fragmentation or surface-induced nucleation dominates in the system, respectively, while $C_{\pm} = \pm\frac{\lambda^2}{2\kappa^2}$ and $D = \frac{\lambda^2}{\kappa^2}$, where $\lambda = \sqrt{2k_nk_+m(0)^{n_C}}$.[1-4]

For a given chaperone-protein system, the aggregation profiles in the absence and presence of n different cheperone concentrations C_i were simulated according to Supplementary Eq.(4) by modifying the microscopic rate constants, which were determined by fitting the experimenal data by minimizing a least-squared error function defined as $y = \sum_{i=1}^{t_{exp}}(M_{sim}(t_i) - M_{exp}(t_i))^2$, where $M_{sim}(t_i)$ and $M_{exp}(t_i)$ are the simulated and the experimental total fibril mass fraction at time t_i, respectively. The same procedure was followed to estimate the association and dissociation rate constants based on the equations described in the following paragraphs, by globally fitting the reaction profiles at n different concentrations of chaperone. In this case, the least-squared error function is defined as: $y = \sum_{j=1}^{n}\sum_{i=1}^{t_{exp}}(M_{sim,j}(t_i) - M_{exp,j}(t_i))^2$, where $M_{sim,j}(t_i)$ and $M_{exp,j}(t_i)$ are, respectively, the simulated and the experimental total fibril mass fraction at time t_i at a given concentration j of molecular chaperone.

Kinetic model in the presence of binding between the molecular chaperone and different protein species

Binding to monomer

In the case of binding between chaperone and monomers (Supplementary Figure 1A), the mass balance equations for the concentrations of soluble monomers ($m(t)$), bound monomers ($m_{\text{bound}}(t)$) and free binding sites of the molecular chaperone ($C_i(t)$) read:

$$\frac{dm(t)}{dt} = -n_Ck_nm(t)^{n_C} - n_2k_2M(t)m(t)^{n_2} - 2k_+m(t)P(t) - k_{\text{on}}m(t)C_i(t) + k_{\text{off}}m_{\text{bound}}(t) \quad (5)$$

$$\frac{dm_{\text{bound}}(t)}{dt} = -\frac{dC_i(t)}{dt} = k_{\text{on}}m(t)C_i(t) - k_{\text{off}}m_{\text{bound}}(t), \quad (6)$$

where $m_{bound}(t)$ is the concentration of soluble monomer and $M(t)$ is the concentration of monomer units incorporated into the fibrils, C_i is the concentration of the free binding sites of the molecular chaperone and k_{on} and k_{off} represent the association and dissociation rate constants. This equation can be integrated together with the mass balance of the total monomer concentration ($m_{\text{tot}}(t)$):

$$m_{tot}(t) = m(t) + m_{bound}(t) + M(t) \quad (7)$$

The binding of the chaperones to the monomer reduces the concentration of reactive soluble monomer ($m(t)$), therefore reducing the primary and secondary nucleation as well as the elongation processes.

Binding to fibril ends

In the case of binding of the chaperone to the fibrils ends (Supplementary Figure 1B), it is convenient to differentiate between three different populations of fibrils in the reaction scheme: in addition to the population of fibrils with no end bound (f), the binding events will generate a population of fibrils with one end bound (f^*) and a population of fibrils with both ends bound (f^{**}). It is important to keep track of the distribution of the filaments among the different populations to quantify the total number of fibril ends, which governs the elongation rate and therefore the rate of fibril growth. On this purpose, we calculate the exchange of fibrils among the populations due to binding and fragmentation events. The breakage of filaments with one end blocked (f^*) generates one shorter fragment in the population with no ends bound (f) and a second fragment which remains in the same population f^*, while the breakage of a fibril with both ends bound (f^{**}) generates two shorter fragments in the population of fibrils with one end bound (f^*). We describe the kinetics of this process by deriving the master equations for the concentration of filaments with a given length j of the three populations.

$$\begin{aligned}\frac{\partial f_j(t)}{\partial t} =& + \delta_{j,n_C} k_n m(t)^{n_C} + \delta_{j,n_2} k_2 m(t)^{n_2} \sum_{i=n_C}^{\infty} i\,(f_i(t) + f_i^*(t) + f_i^{**}(t)) \\ &+ 2k_+ m(t)(f_{j-1}(t) - f_j(t)) \\ &- k_-(j-1) f_j(t) + 2k_- \sum_{i=j+1}^{\infty} f_i(t) + k_- \sum_{i=j+1}^{\infty} f_i^*(t) \\ &- k_{onEnd} f_j(t) C_i(t) + k_{offEnd} f_j^*(t)\end{aligned} \tag{8}$$

$$\begin{aligned}\frac{\partial f_j^*(t)}{\partial t} =& + k_+ m(t)(f_{j-1}^*(t) - f_j^*(t)) \\ &- k_-(j-1) f_j^*(t) + k_- \sum_{i=j+1}^{\infty} f_i^*(t) + 2k_- \sum_{i=j+1}^{\infty} f_i^{**}(t) \\ &+ k_{onEnd} f_j(t) C_i(t) - k_{offEnd} f_j^*(t) \\ &- k_{onEnd} f_j^*(t) C_i(t) + k_{offEnd} f_j^{**}(t)\end{aligned} \tag{9}$$

$$\frac{\partial f_j^{**}(t)}{\partial t} = - k_-(j-1) f_j^{**}(t) + k_{onEnd} f_j^*(t) C_i(t) - k_{offEnd} f_j^{**}(t) \tag{10}$$

where k_{onEnd} and k_{offEnd} are the association and dissociation rate constants, respectively, and $C_i(t)$ is the concentration of the molecular chaperone. The master equations can be solved by

applying the method of the moments and deriving the mass balance equations for the total fibril concentration of the different populations:

$$\begin{aligned}\frac{\partial M(t)}{\partial t} =& n_C k_n m(t)^{n_C} + n_2 k_2 \left(M(t) + M^*(t) + M^{**}(t)\right) m(t)^{n_2} \\ &+ 2k_+ m(t)P(t) + k_- \left(\frac{Q(t)^*}{2} - \frac{M(t)^*}{2}\right) - k_{onEnd} M(t) C_i(t) + k_{offEnd} M^*(t)\end{aligned} \tag{11}$$

$$\begin{aligned}\frac{\partial M^*(t)}{\partial t} =& k_+ m(t) P^*(t) - k_- \left(\frac{Q(t)^*}{2} - \frac{M(t)^*}{2}\right) + k_-(Q(t)^{**} - M(t)^{**}) \\ &+ k_{onEnd} M(t) C_i(t) - k_{offEnd} M^*(t) - k_{onEnd} M^*(t) C_i(t) + k_{offEnd} M^{**}(t)\end{aligned} \tag{12}$$

$$\frac{\partial M^{**}(t)}{\partial t} = -k_-(Q(t)^{**} - M(t)^{**}) + k_{onEnd} M^*(t) C_i(t) - k_{offEnd} M^{**}(t) \tag{13}$$

$$\begin{aligned}\frac{\partial P(t)}{\partial t} =& k_n m(t)^{n_C} + k_2 \left(M(t) + M^*(t) + M^{**}(t)\right) m(t)^{n_2} + k_- \left(M(t) + (1 - 2n_C P(t))\right) \\ &+ k_- \left(M^*(t) - n_C P^*(t))\right) - k_{onEnd} P(t) C_i(t) + k_{offEnd} P^*(t)\end{aligned} \tag{14}$$

$$\begin{aligned}\frac{\partial P^*(t)}{\partial t} =& + k_- \left((1 - n_C) P^*(t))\right) + 2k_- \left(M^{**}(t) - n_C P^{**}(t))\right) \\ &+ k_{onEnd} P(t) C_i(t) - k_{offEnd} P^*(t) - k_{onEnd} P^*(t) C_i(t) + k_{offEnd} P^{**}(t)\end{aligned} \tag{15}$$

$$\frac{\partial P^{**}(t)}{\partial t} = k_{onEnd} P^*(t) C_i(t) - k_{offEnd} P^{**}(t) + k_- \left(-M^{**}(t) + P^{**}(t))\right) \tag{16}$$

$$\begin{aligned}\frac{\partial Q(t)}{\partial t} =& n_C^2 k_n m(t)^{n_C} + n_2^2 k_2 \left(M(t) + M^*(t) + M^{**}(t)\right) m(t)^{n_2} + 2k_+ m(t) \left(2M(t) + P(t)\right) \\ &+ k_- \left(-\frac{5T(t)}{3} + \frac{M(t)}{3}\right) + k_- \left(-\frac{T(t)^*}{3} - \frac{Q(t)^*}{2} + \frac{M(t)^*}{6}\right) \\ &- k_{onEnd} Q(t) C_i(t) + k_{offEnd} Q^*(t)\end{aligned} \tag{17}$$

$$
\begin{aligned}
\frac{\partial Q^*(t)}{\partial t} =& k_{onEnd}Q(t)C_i(t) - k_{offEnd}Q^*(t) - k_{onEnd}Q^*(t)C_i(t) + k_{offEnd}Q^{**}(t) \\
&+ k_+m(t)\left(2M(t)^* + P(t)^*\right) + k_-\left(-T(t)^* + Q(t)^*\right) + k_-\left(-\frac{T(t)^*}{3} - \frac{Q(t)^*}{2} + \frac{M(t)^*}{6}\right) \\
&+ 2k_-\left(-\frac{T(t)^{**}}{3} - \frac{Q(t)^{**}}{2} + \frac{M(t)^{**}}{6}\right)
\end{aligned} \tag{18}
$$

$$
\frac{\partial Q^{**}(t)}{\partial t} = k_{onEnd}Q^*(t)C_i(t) - k_{offEnd}Q^{**}(t) + k_-\left(-T^{**}(t) + Q^{**}(t)\right)) \tag{19}
$$

where P, M and Q represent respectively the zero, first and second order moment of the corresponding fibril population. Considering a gamma distribution, the moment of the third order, T, can be calculated according to the closure equation:

$$
T = \frac{Q\left(2QP - M^2\right)}{MP} \tag{20}
$$

It is worth verifying that the time evolution of the total mass: $M_{tot}(t) = M(t) + M^*(t) + M^{**}(t)$ depends only on the nucleation and the elongation events:

$$
\begin{aligned}
\frac{\partial M_{tot}(t)}{\partial t} =& \frac{\partial M(t)}{\partial t} + \frac{\partial M^*(t)}{\partial t} + \frac{\partial M^{**}(t)}{\partial t} = \\
& n_C k_n m(t)^{n_C} + n_2 k_2 M_{tot}(t) m(t)^{n_2} + 2k_+m(t)P(t) + k_+m(t)P^*(t)
\end{aligned} \tag{21}
$$

since the binding and breakage events exchange filaments among the different populations but do not modify the total fibrillar mass. The binding of the inhibitor to the fibril ends reduces the elongation rate, which in the absence of inhibitor is equal to $r_+ = 2k_+m(t)P_{tot}(t)$, since now only a fraction of the total number of fibrils equal to P/P_{tot} can propagate from both ends, and another fraction of fibril equal to P^*/P_{tot} can react only from one end. The effective elongation rate becomes $r_+ = 2k_+m(t)P(t) + k_+m(t)P^*(t)$.

Binding on fibril surface

The binding of the chaperone along the fibril surfaces (Supplementary Figure 1C) decreases the number of monomeric units incorporated in the fibrils which are able to catalyze secondary nucleation events. The amount of monomeric units which is deactivated, M_{bound}, can be calculated by considering a Langmuir-type adsorption of the chaperone on the fibril surface, which in its simplest form assumes independent binding events on different sites. The binding rate is proportional to the concentration of free sites and the concentration of soluble chaperone:

$$
\frac{\partial M_{bound}(t)}{\partial t} = k_{onSurf}C_i(t)\big(M_{tot}(t)\sigma - M_{bound}(t)\big) - k_{offSurf}M_{bound}(t) \tag{22}
$$

The parameter σ describes the fraction between the number of available binding sites and the total number of monomeric units in the filaments: σ is equal to 1 when one chaperone molecule can bind each single monomeric unit, and is lower than 1 when the number of binding sites is lower than the number of monomers. The secondary nucleation reaction rate is proportional to the number of unbound monomeric units in the fibrils: $r_2 = k_2(M(t) - \theta M_{bound}(t))m(t)^{n_2}$. The parameter θ is the overlap-site size, which describes the number of monomeric units occluded by a single bound ligand. This parameter is larger than one if the presence of the chaperone on the surface decreases the activity not only of the bound monomeric unit but also of the neighbour monomeric units. The mass balance for the total fibril concentration in the presence of binding of the inhibitor along the fibril surface is modified as follows:

$$\frac{\partial M(t)}{\partial t} = n_C k_n m(t)^{n_C} + n_2 k_2 \big(M(t) - \theta M_{bound}(t)\big) m(t)^{n_2} + 2k_+ m(t) P(t) \tag{23}$$

Depending on the specific system under investigation, all or only some of the mechanisms described above may be relevant. Supplementary Eqs (5)-(23) must be coupled with the mass balance of the chaperone, which in the most general case is:

$$C_i^{Tot} = C_i^{Sol} + C_i^{Mon} + C_i^{FibrilEnd} + C_i^{FibrilSurf} \tag{24}$$

where C_i^{Tot} is the total chaperone concentration, C_i^{Sol} is the concentration of the soluble chaperone and C_i^{Mon},$C_i^{FibrilEnd}$ and $C_i^{FibrilSurf}$ represent the concentration of chaperone bound to monomers, fibril ends and fibril surface, respectively.

Simplified expressions and quantification of interaction energies from reaction rates

In the most generic case, the rate of generation of protein species which interact with the inhibitor can be comparable to the binding rate. As a consequence, the binding events cannot be considered a priori at equilibrium with respect to the aggregation processes, and the set of Supplementary Eqs (5)-(23) must be solved numerically. However, in some limiting situations, compact expressions of the inhibited microscopic rate constants as a function of the equilibrium binding constant can be derived, in particular in the situations where the inhibitor is present in excess with respect to the protein species and the binding reaction can be considered at equilibrium. In this case, we can derive apparent rate constants which are analogous to the closed expressions obtained in the context of enzyme inhibition in the presence of ligands,[6] where the measured apparent values of specificity and catalytic rate constants can be easily expressed as function of the ligand concentration and the binding equilibrium constant. The analysis of the dependence of the rate constants on the ligand concentration provides information on the enzyme inhibition mechanism, such as the presence of competitivity and cooperativity.[6] Approximative closed-form expressions for the reaction time course, are discussed in the next section.

Binding to monomer

In the case of binding to monomers, it is trivial to derive from the mass balance of the total monomer concentration the expressions for the soluble unbound monomer concentration and the effective microscopic rate constants as a function of the binding equilibrium constant:

$$m(t) = m(t)_{sol} + m(t)_{bound} \tag{25}$$

$$m(t)_{sol} = \frac{m(t)}{1 + K_{eqMon} C_i^{Tot}} \tag{26}$$

$$k_N^{app} = k_N \frac{1}{(1 + K_{eqMon} C_i^{Tot})^{n_C}} \tag{27}$$

$$k_+^{app} = k_+ \frac{1}{1 + K_{eqMon} C_i^{Tot}} \tag{28}$$

$$k_2^{app} = k_2 \frac{1}{(1 + K_{eqMon} C_i^{Tot})^{n_2}} \tag{29}$$

Binding to fibril ends

Similarly, in the case of binding to fibril-ends the following expression for the concentration of unbound soluble fibril-ends and for the elongation rate constant can be derived:

$$P^{Tot}(t) = P(t) + P^*(t) + P^{**}(t) \tag{30}$$

$$P^{**}(t) = K_{eqEnd} C_i^{Tot} P^*(t) \tag{31}$$

$$P^*(t) = K_{eqEnd} C_i^{Tot} P(t) \tag{32}$$

$$P(t) = \frac{P^{Tot}(t)}{1 + K_{eqEnd} C_i^{Tot} + (K_{eqEnd} C_i^{Tot})^2} \tag{33}$$

$$k_+^{app}/k_+ = \frac{2P(t) + P^*(t)}{2P^{Tot}(t)} = \frac{2 + K_{eqEnd} C_i^{Tot}}{2 + 2K_{eqEnd} C_i^{Tot} + 2\left(K_{eqEnd} C_i^{Tot}\right)^2} \tag{34}$$

Binding on fibril surface

Finally, an analogous expression for the secondary nucleation rate constant can be derived in the case of binding along fibril surface:

$$M_{bound}(t) = K_{eqSurf} C_i^{Tot} M(t) \tag{35}$$

$$k_2^{app} = k_2 \left(1 - K_{eqSurf} C_i^{Tot}\right) \tag{36}$$

An example of a situation where these simplied expressions can be applied is represented by the Ure2p-Hsp70 system discussed in the main text. In Supplementary Figure 2a we compare

the simulated reaction rates for nucleation ≈ generation of new fibril ends ($r_2(t) \approx k_-M(t)$), elongation, ($r_+(t) = 2k_+P(t)m(t)$), and binding, ($r_B(t) = k_{on}M(t)C_i(t)$) corresponding to the simulated reaction profiles of the Ure2p-Hsp70 system shown in Figure 2e in the main text. The rates are calculated at the reference protein-chaperone ratio 1:0.5. In this case, the association and dissociation rates are several orders of magnitude larger than the secondary nucleation rate by fibril fragmentation. The association and dissociation events are therefore much faster than the generation of new fibril ends by breakage events, and the binding reaction can be considered at equilibrium with respect to the aggregation process. In this case, the effective elongation rates can be described by Supplementary Eq.(26).

This observation explains why the semi-empirical approach based on Eq.1 in the main text can describe the global kinetic profiles by considering a single effective apparent elongation rate constant during the overall process. In Supplementary Figure 2b we compare the apparent values estimated by the fitting shown in Figure 2 in the main text with the effective elongation rate constants predicted by Supplementary Eq.(26). The two sets of values are in excellent agreement and, as expected, the value of K_{eq} required to describe the apparent elongation rate constants with Supplementary Eq.(26) is equal to the equilibrium constant evaluated by the integrated rate laws shown in Figure 2. This observation highlights the fact that, in systems where the binding process can be considered to proceed under equilibrium conditions, the apparent kinetic constants evaluated by the semi-empirical approach based on Eq.1 in the main text can provide a direct measurement of the equilibrium binding constant (K_{eq}) and of the free energy of binding ($\triangle G^0_{binding} = -RTlnK_{eq}$).

Analytical closed-form solutions for fibril growth in the presence of binding between the molecular chaperone and monomers

In this section, we sketch out a general mathematical treatment for obtaining closed-form solutions for fibril growth in the presence of chaperone binding to the monomers; the cases when molecular chaperones bind fibril ends or surface can be treated using a similar approach. The starting point of our treatment of filamentous growth kinetics with chaperone binding to monomers is given by the following kinetic equations

$$\frac{dP(t)}{dt} = k_n m(t)^{n_C} + k_2 M(t) m(t)^{n_2} \tag{37}$$

$$\frac{dm(t)}{dt} = -2k_+ m(t)P(t) - k_{\text{on}} m(t) C_i(t) + k_{\text{off}} m_{\text{bound}}(t) \tag{38}$$

$$\frac{dm_{\text{bound}}(t)}{dt} = -\frac{dC_i(t)}{dt} = k_{\text{on}} m(t) C_i(t) - k_{\text{off}} m_{\text{bound}}(t), \tag{39}$$

where k_2 is the rate constant for the secondary nucleation process and n_2 defines the dependence of this process on the concentration of free monomers, with $n_2 = 0$ corresponding to fragmentation, $n_2 = 1$ corresponding to lateral branching and $n_2 \geq 2$ describing surface catalysed secondary nucleation. Note that in Supplementary Eq. (38) we have neglected the terms $-n_C k_n m(t)^{n_C}$ and $-n_2 k_2 m(t)^{n_2} M(t)$ describing the depletion of monomers through nucleation processes. This set

of equations can be most conveniently written in the dimensionless form

$$\frac{d\Pi(\tau)}{d\tau} = \nu_1 \mu(\tau)^{n_C} + \nu_2[1 - \mu(\tau) - \mu_{\text{b}}(\tau)]\mu(\tau)^{n_2} \tag{40}$$

$$\frac{d\mu(\tau)}{d\tau} = -\mu(\tau)\Pi(\tau) - \beta\mu(\tau)\gamma_i(\tau) + \alpha\mu_{\text{b}}(\tau) \tag{41}$$

$$\frac{d\mu_{\text{b}}(\tau)}{d\tau} = -\frac{d\gamma_i(\tau)}{d\tau} = \beta\mu(\tau)\gamma_i(\tau) - \alpha\mu_{\text{b}}(\tau), \tag{42}$$

where we have introduced the following rescaled variables $\tau = 2k_+ m_{\text{tot}} t$, $\mu(\tau) = m(\tau)/m_{\text{tot}}$, $\mu_b(\tau) = m_{\text{bound}}(\tau)/m_{\text{tot}}$, $\gamma_i(\tau) = C_i(\tau)/m_{\text{tot}}$, $\Pi(\tau) = P(\tau)/m_{\text{tot}}$ and the following parameters

$$\nu_1 = \frac{k_n m_{\text{tot}}^{n_C - 2}}{2k_+}, \quad \nu_2 = \frac{k_2 m_{\text{tot}}^{n_2 - 1}}{2k_+}, \quad \alpha = \frac{k_{\text{off}}}{2k_+ m_{\text{tot}}}, \quad \beta = \frac{k_{\text{on}}}{2k_+}. \tag{43}$$

The parameters ν_1, ν_2, α and β defined in Supplementary Eq. (43) are dimensionless combinations of the rate parameters that describe the relative importance of nucleation and binding processes to filament elongation. Thus, under typical environmental conditions $\nu_1 \ll 1$, such that we seek a solution of Supplementary Eqs. (40)-(42) in form of a perturbation series in the (small) parameter ν_1:

$$\Pi(\tau) = \Pi_0(\tau) + \nu_1 \Pi_1(\tau) + \nu_1^2 \Pi_2(\tau) + \cdots \tag{44}$$

$$\mu(\tau) = \mu_0(\tau) + \nu_1 \mu_1(\tau) + \nu_1^2 \mu_2(\tau) + \cdots \tag{45}$$

$$\mu_b(\tau) = \mu_{b,0}(\tau) + \nu_1 \mu_{b,1}(\tau) + \nu_1^2 \mu_{b,2}(\tau) + \cdots \tag{46}$$

$$\gamma_i(\tau) = \gamma_{i,0}(\tau) + \nu_1 \gamma_{i,1}(\tau) + \nu_1^2 \gamma_{i,2}(\tau) + \cdots \tag{47}$$

After solving Supplementary Eqs. (40)-(42) separately for each order of ν_1, and applying the initial condition $\mu(0) = 1$, we find the following expansion at order $\mathcal{O}(\nu_1)$

$$\mu(\tau) + \mu_b(\tau) = 1 - \frac{\nu_1(1-\lambda_2)^{n_C - n_2}}{2\nu_2}\left(e^{\sqrt{\nu_2(1-\lambda_2)^{n_2+1}}\tau} + e^{-\sqrt{\nu_2(1-\lambda_2)^{n_2+1}}\tau} - 2\right), \tag{48}$$

where

$$\lambda_2 = \frac{1}{2}\left(1 + \gamma_0 + \frac{\alpha}{\beta} - \sqrt{\left(1 + \gamma_0 + \frac{\alpha}{\beta}\right)^2 - 4\gamma_0}\right) \tag{49}$$

Using the perturbative renormalization group approach[7-8] to remove the secular divergent term in Supplementary Eq. (48), yields the following result for the fibril mass concentration:

$$\frac{M(t)}{m_{\text{tot}}} = 1 - \exp\left(-\tilde{C}_+ e^{\tilde{\kappa}t} + \tilde{C}_- e^{-\tilde{\kappa}t} + \tilde{D}\right), \tag{50}$$

where the dominant kinetic parameter is given by $\tilde{\kappa} = \sqrt{2m(0)k_2k_+m(0)^{n_2}(1-\lambda_2)^{n_2+1}}$, while $\tilde{C}_\pm = \pm\frac{\tilde{\lambda}^2}{2\tilde{\kappa}^2}$ and $\tilde{D} = \frac{\tilde{\lambda}^2}{\tilde{\kappa}^2}$, where $\tilde{\lambda} = \sqrt{2m(0)k_nk_+m(0)^{n_C}(1-\lambda_2)^{n_C+1}}$.

In the cases where the dominant secondary mechanism is monomer-dependent secondary nucleation ($n_2 \geq 1$), a more accurate expression of Supplementary Eq. 50 can be obtained, which reads:

$$\frac{M(t)}{m_{tot}} = 1 - \left[1 + \frac{\tilde{\lambda}^2}{2\theta\tilde{\kappa}^2} e^{\tilde{\kappa}t}\right]^{-\theta} \tag{51}$$

where $\theta = \sqrt{2/[n_2(n_2+1)]}$.

The analytical close-form solutions were validated by comparison with numerical calculations. In Supplementary Figure 3 we show this comparison for the two systems discussed in the main text: the Aβ42 peptide and the Brichos chaperone, and the Ure2p and the Ssa1 chaperone.

Supplementary References

1. Cohen, S.I.A., Vendruscolo, M., Welland, M.E., Dobson, C.M., Terentjev, E.M. & Knowles, T.P.J. Nucleated polymerization with secondary pathways. I. Time evolution of the principal moments. *J. Chem. Phys.* **135**, 065105 (2011)

2. Cohen, S.I.A., Vendruscolo, M., Dobson, C.M. & Knowles, T.P.J. Nucleated polymerization with secondary pathways. II. Determination of self-consistent solutions to growth processes described by non-linear master equations. *J. Chem. Phys.* **135**, 065106 (2011)

3. Cohen, S.I.A., Vendruscolo, M., Dobson, C.M. & Knowles, T.P.J. Nucleated polymerization with secondary pathways. III. Equilibrium behavior and oligomer populations. *J. Chem. Phys.* **135**, 065107 (2011)

4. Knowles, T.P.J. *et al.* An Analytical Solution to the Kinetics of Breakable Filament Assembly. *Science* **326**, 1533-1537 (2009).

5. Cohen, S.I.A. *et al.* Proliferation of Abeta42 aggregates occurs through a secondary nucleation mechanism. *Proc. Natl Acad. Sci. USA* **110** , 97589763 (2013).

6. Segel, I.H., *Enzyme Kinetics: Behavior and Analysis of Rapid Equilibrium and Steady-State Enzyme Systems*, Wiley Classis Library, (1993)

7. Chen, L.Y., Goldenfeld, N. & Oono Y. Renormalization group and singular perturbations: Multiple scales, boundary layers, and reductive perturbation theory. *Phys. Rev. E* **54**, 376-394 (1996).

8. Michaels, T.C.T. & Knowles, T.P.J. Kinetic theory of protein filament growth: Self-consistent methods and perturbative techniques. *Int. J. Mod. Phys. B* **29**, 1530002 (2015).
